# MiR-886-3p Down Regulates CXCL12 (SDF1) Expression in Human Marrow Stromal Cells

**DOI:** 10.1371/journal.pone.0014304

**Published:** 2010-12-13

**Authors:** Manoj M. Pillai, Xiaodong Yang, Ilango Balakrishnan, Lynne Bemis, Beverly Torok-Storb

**Affiliations:** 1 Division of Medical Oncology, Department of Medicine, University of Colorado Denver, Aurora, Colorado, United States of America; 2 Clinical Research Division, Fred Hutchinson Cancer Research Center, Seattle, Washington, United States of America; City of Hope, United States of America

## Abstract

Stromal Derived Factor 1 (SDF1 or CXCL12), is a chemokine known to be critical for the migration of cells in several tissue systems including the homing of the hematopoietic stem cell (HSC) to its niche in the bone marrow. A comparative analysis of miRNA expression profiles of two stromal cell lines, distinguishable by function and by CXCL12 expression (CXCL12^+^ and CXCL12^−^), revealed that the CXCL12^−^ cells expressed >40 fold more miR-886-3p than the CXCL12^+^ cells. Screening studies showed that when miR-886-3p was transfected into the CXCL12^+^ stromal cells, the expression of CXCL12 was down-regulated by as much as 85% when compared to appropriate controls, and results in the loss of CXCL12-directed chemotaxis. Similar reductions in CXCL12 were obtained with the transfection of miR-886-3p into primary stromal cell cultures. Additional studies showed that miR-886-3p specifically targeted the 3′ untranslated region (UTR) of CXCL12 mRNA. These data suggest a role for miRNA in modulating the expression of CXCL12, a gene product with a critical role in hematopoietic regulation.

## Introduction

SDF1 or CXCL12 is a chemokine which binds specifically to the G-protein coupled receptor, CXCR4 [1]. Although originally isolated from murine marrow stromal cells, CXCL12 is now known to be expressed in several cell types, as is its cognate receptor, CXCR4. In vitro and in vivo studies have now established the critical role CXCL12/CXCR4 interactions play in the directed migration of cells within tissues. In the hematopoietic system, stem and progenitor cells express CXCR4 and migrate to their niches along a gradient of CXCL12 expressed by the cellular components of the niche[2],[3]. Neuronal migration during development utilizes this ligand-receptor system [4] as do tumor cells metastasizing from their primary site to their metastatic niches [5]. Knock-out mice lacking either the ligand or the receptor are embryonic lethal, with defects in multiple organs [4], [6].

In the hematopoietic system, CXCL12 is now known to be amongst several factors that contribute to a functional stem cell niche [3]. Other stem-cell niche defining genes identified in the past several years include Jagged1[7], Angiopoietin1[8], BMP4[9], Osteopontin[10], and N-Cadherin[11]. How the cellular elements in the niche express these factors in a coordinated fashion is not known. For instance, CXCL12 is down-regulated by cytokines such as IL1 [12] and FGF2 [13], but this appears to be promoter-independent. We hypothesized that trans-regulatory factors such as transcription factors and/or microRNAs (miRNAs) are likely involved in coordinating the expression of CXCL12. We further hypothesized that stromal cell lines that differed in CXCL12 expression could be used in a comparative analysis to identify differentially expressed miRNAs that may be responsible for controlling CXCL12 expression.

We have previously reported on two stromal cell lines, derived from one human primary long term culture, that have distinguishable functional characteristics and gene expression profiles [14]. One termed HS27a expresses high levels of CXCL12 and niche-associated genes and functions to support primitive hematopoietic precursors in specialized areas of culture called cobble-stone areas. In contrast, a second line termed HS5 expresses low levels niche-associated ligands, but secretes high levels of IL1, IL6 and GCSF, factors which drive hematopoietic precursors to differentiate and proliferate. Consequently HS5 also does not support formation of cobble stone areas. Using miRNA microarrays, we identified miR-886-3p to be expressed 40 fold greater in HS5 compared to HS27a. Interestingly, the primary transcript of miR-886-3p was recently reported to be the RNA component of a ribonucleoprotein called vault [15]. In this report we show that miR-886-3p directly targets the 3′UTR of CXCL12 mRNA, significantly down-regulating the expression and function of CXCL12. The ability of miR-866-3p to decrease CXCL12 expression was also observed in primary stromal cells derived from long term marrow cultures and in non-stromal cancer cell lines known to express CXCL12.

## Results

### Human Stromal Cell line HS27a expresses HSC niche–associated ligands

**Figure 1:**
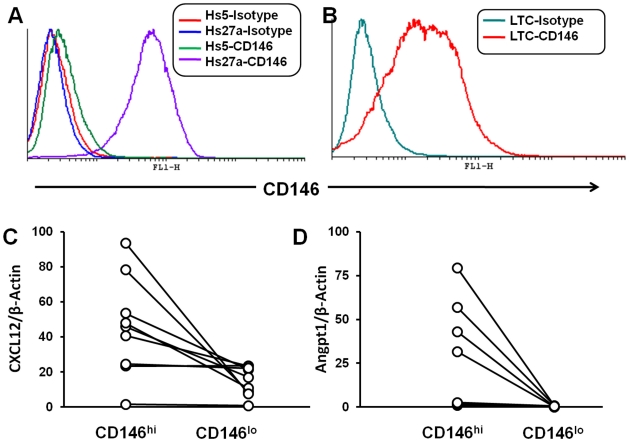
Expression of CD146, CXCL12 and Angiopoietin 1 in stromal cells and primary long term cultures (LTCs). A: FACS analysis of stromal cell lines HS5 and HS27a for CD146 (MCAM) along with isotype controls. B: FACS analysis of a primary LTC at first passage for CD146 along with isotype control. The primary LTCs were sorted to CD146 ^hi^ and CD146 ^lo^ populations by FACS-aided flow-sorting. Approximate gates for hi and lo populations are also indicated. C: CXCL12 levels of 9 LTCs sorted to CD146^hi^ and CD146^lo^ and quantitated by RT-PCR (relative to beta-actin, multiplied by a factor of 1000). Paired samples from the same culture are connected by a line. D: Angiopoietin-1 levels of the same 9 LTCs, also quantitated by RT-PCR (relative to beta-actin, multiplied by a factor of 10000). Experiment in panel A was confirmed in three independent experiments, Experiments in panels B, C and D were confirmed in 9 independent samples. Statistical analysis of the datasets in Panels C and D by Wilcoxon's matched pair test revealed a p-value of <0.01 in both cases.

Mesenchyme-derived stromal fibroblasts (also referred to as marrow stromal cells, mesenchymal stem cells, or MSC) are now known to play a critical role in the regulation of hematopoiesis as defined by both in vivo and in vitro models [16]. Although the term MSC has come to imply that these cells are homogenous, it is now recognized that the MSC population contains subsets with distinct functions, although isolating these subsets by surface labeling has been difficult. However, recently the expression of CD146 (MCAM or Melanoma Cell Associated Molecule) has been reported to define a subset of stromal precursors that contribute to the stem cell niche [17]. We speculated that since the HS27a stromal cell line seemed to share similar functional and transcriptional profiles with those reported for the CD146^hi^ stromal precursors, it might also express high levels of CD146. We performed flow-cytometric analysis of CD146 on HS27a and HS5 cells and determined that CD146 was expressed strongly by HS27a, whereas HS5 did not (**Figure 1A**). We then evaluated CD146 expression in 9 primary cultures, established from normal bone marrow. The cultures which were analyzed within the first two to three passages demonstrated variable proportions of CD146^hi^ cells (6 to 40%), an example of which is shown in **Figure 1B**. The CD146^hi^ and CD146^lo^ cells from primary cultures were then evaluated for expression of two genes deemed critical to the stem cell niche, CXCL12 and Angiopoietin 1. As shown in **Figures 1C and 1D**, the CD146^hi^ population has significantly higher expression of both CXCL12 and Angiopoietin 1 when compared to CD146^lo^ cells. Thus CD146^hi^ stromal cells from primary cultures retain the expression profile associated with the HSC niche. HS27a also expresses these niche associated genes; this together with its functional phenotype makes it a relevant model for at least one cellular component of the HSC niche.

### The stromal cell lines HS5 and HS27a have distinct miRNA expression profiles

To test our hypothesis that miRNAs may influence the expression levels of niche-associated genes, we first determined the miRNA expression profiles of the functionally distinct HS27a and HS5 cell lines. 1×10^6^ cells of each cell line, in log-phase growth were harvested and total RNA including the small non-coding fraction were isolated and submitted for miRNA analysis. Of the 810 human miRNAs arrayed, 156 had statistically different expression levels in the two cell lines. **Table 1** provides a partial list of 10 miRNAs over-expressed in HS27a when compared to HS5, and **Table 2** provides a list of miRNAs over expressed in HS5 when compared to HS27a. The complete data-set is available in the geo omnibus database as well in **Table S1**. The 48-fold over-expression of miR-886-3p in HS27a was of interest given that such a profound expression difference was likely to have functional consequences.

**Table 1:** miRNAs over-expressed in HS27a.

Sanger ID	Accession Number	Ratio (HS27a/HS5)	p value
hsa-miR-181a	MIMAT0000256	4.96	0.01
hsa-miR-21	MIMAT0000076	2.92	0.01
hsa-miR-181b	MIMAT0000257	2.38	<0.01
hsa-miR-224	MIMAT0000281	2.30	0.01
hsa-miR-21*	MIMAT0004494	2.23	0.01
hsa-miR-7	MIMAT0000252	2.17	0.01
hsa-miR-299-5p	MIMAT0002890	1.79	0.01
hsa-miR-31	MIMAT0000089	1.77	0.01
hsa-miR-25	MIMAT0004498	1.75	0.01
hsa-miR-155	MIMAT0000646	1.67	<0.01

Partial list of miRNAs significantly over-expressed in HS27a when compared to HS5. Three independent samples were analyzed for microRNA expression profile.

**Table 2:** miRNAs over-expressed in HS5.

Sanger ID	Accession Number	Ratio(HS5/HS27a)	p value
hsa-miR-886-3p	MIMAT0004906	48.49	<0.01
hsa-miR-9	MIMAT0000441	2.97	0.01
hsa-miR-193a-3p	MIMAT0000459	2.93	<0.01
hsa-miR-365	MIMAT0000710	2.52	0.02
hsa-miR-210	MIMAT0000267	2.31	0.04
hsa-miR-26b	MIMAT0004500	2.28	<0.01
hsa-miR-146a	MIMAT0000449	2.10	0.04
hsa-miR-222	MIMAT0000279	2.10	0.02
hsa-miR-149	MIMAT0000450	2.08	<0.01
hsa-miR-148a	MIMAT0000243	2.01	0.01

Partial list of miRNAs significantly over-expressed in HS5 when compared to HS27a. Three independent samples were analyzed for the microRNA expression profile.

**Figure 2:**
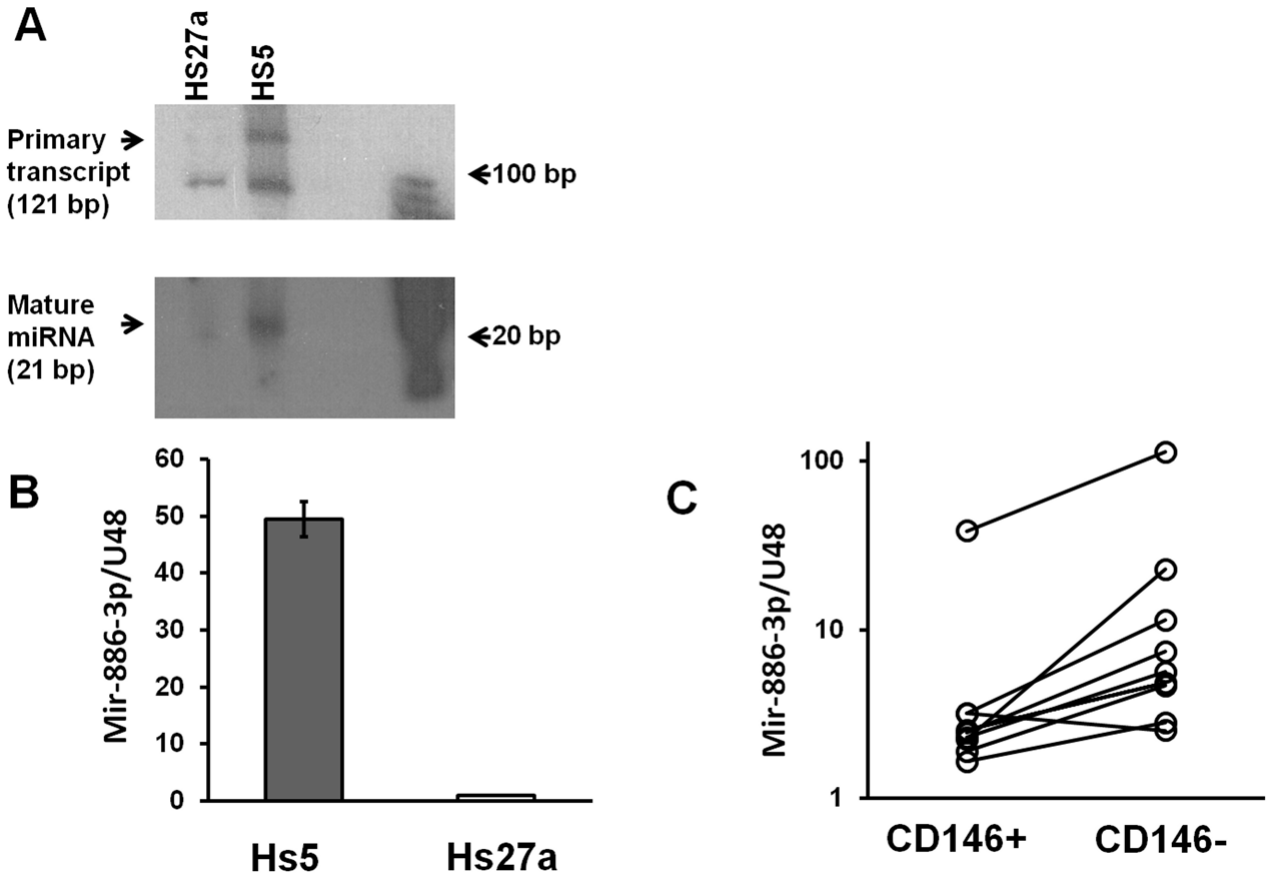
miR-886-3p expression in stromal cells. **A: Northern Blotting for has-miR-886-3p.** The 121 bp primary transcript of miR-886-3p and the 21 bp mature miR-886-3p are detected by by Northern blotting using a probe antisense to mature miR-886-3p (21 nucleotides). Both the primary transcript and mature miRNA are expressed at a higher level in HS5 cells. The primary transcript was visible at lower exposure times (typically ∼4 hours) compared to the mature 21 bp transcript (typically overnight exposure). **B**: miR-886-3p expression in HS5 compared and HS27a, normalized to the expression of U48, a small non-coding RNA. Results are expressed as a ratio of HS5 levels over HS27a levels for three samples shown are mean of three samples, ±SE (Standard Error). **C**: Expression of miR-886-3p in CD146 high and CD146 low populations of 9 primary long-term cultures (LTCs) in early passages, also determined by q RT PCR. Paired samples are connected by a line. Statistical analysis was performed by Wilcoxon matched pair test, a non-parametric test using GraphPad Prism software, which shows a p-value <0.01.

MiR-886-3p has been described in both humans [18] and macaques [19] by deep sequencing of small RNA libraries, but it has not been detected by size fractionating (such as by Northern blotting). Interestingly, the primary transcript of miR-886-3p is now thought to be a component of vaults (a ribonucleoprotein particle) and has been annotated vtRNA-2[15]. It has been suggested that miR-886-3p might not be a microRNA [20], however as shown in **Figure 2A**, both the 121 nucleotide primary transcript and the 21-nucleotide miR-886-3p are detectable by northern blotting with higher levels in HS5 cells compared to HS27a. This confirms that the 21 nucleotide long miR-886-3p is indeed processed from the primary transcript and hence is a genuine miRNA.

Our results are in agreement with a report by Persson et. al that show other vault RNAs (vtRNA-1) can be processed to smaller functional counterparts through a Drosha-independent but dicer-dependent mechanism [21]. Similarly, small nucleolar RNA (snoRNA) were found to be processed by Dicer to smaller 20-22 nucleotide RNAs with miRNA-like functions [22]. Results from microarray analysis and northern blot were confirmed by quantitative RT-PCR. As shown in **Figure 2B**, HS5 was shown to have a 50-fold higher level of miR-886-3p compared to HS27a. Quantitation of miRNAs was done by a modification of the stem-loop reverse transcription method described by Chen et al.; primers used are listed in **Table S2**. Results were normalized to the expression of a non-coding small RNA, U48. Although miR-886-3p homologues have not been described in non-primates, vault RNAs have been described in all vertebrates including mouse. It is conceivable that functional miRNAs may arise out of processing of this precursor non-coding RNAs. This work is currently on-goin.

### Primary marrow-derived CD146^hi^ stromal cells that express niche associated gene products express lower levels of miR-886-3p

Results suggested that CD146 expression defined a primary stromal cell population that has functional and transcriptome profiles similar to that of HS27a. To extend the comparison we tested whether CD146^hi^ population of primary stromal cells also have a lower expression of miR-886-3p when compared to the CD146^lo^ population. Nine primary stromal cell cultures were harvested before passage three and sorted into CD146 ^hi^ and CD146 ^lo^ populations. Total RNA was prepared from both populations and reverse transcribed with primers specific for miR-886-3p and U48. Quantitative RT-PCR was then performed and analyzed; results are shown in **Figure 2C**. As anticipated, primary LTCs have significant variation in basal expression of miR-886-3p. However, the CD146^hi^ population had a significantly lower expression of miR-886-3p when compared' to the CD146 ^lo^ population. This indicates that primary marrow stromal cells are similar to HS27a in that their expression of CD146 and CXCL12 are associated with relatively low expression levels of miR-886-3p.

### CXCL12 is a target of miR-886-3p

Although HS5 and HS27a were derived from the same culture, they differentially express several genes that define the stem cell niche, as well as miR-886-3p. We hypothesized that miR-886-3p expression may be responsible for some of these differences. We began by analyzing the targets of 886-3p as defined by commonly used prediction algorithms, miRBASE (http://microrna.sanger.ac.uk/sequences/) [23] and miRANDA (http://www.microrna.org/microrna/home.do) [24]. However no predicted matches with any of the “niche-defining” genes were identified. Nevertheless we proceeded with studies to determine the effect of miR-886-3p on select gene expression. This was justified by our understanding that; 1) the prediction algorithms that match miRNAs to potential binding sites in target mRNA are often imperfect, relying on binding site homologies across species; miR-886-3p has been described only in *Homo sapiens* and *Macacus mulata* and hence homology comparisons may not be relevant across several species; and 2) the effect of miR-886-3p on the above genes of interest may be indirect and hence not apparent by target prediction algorithms.

**Figure 3:**
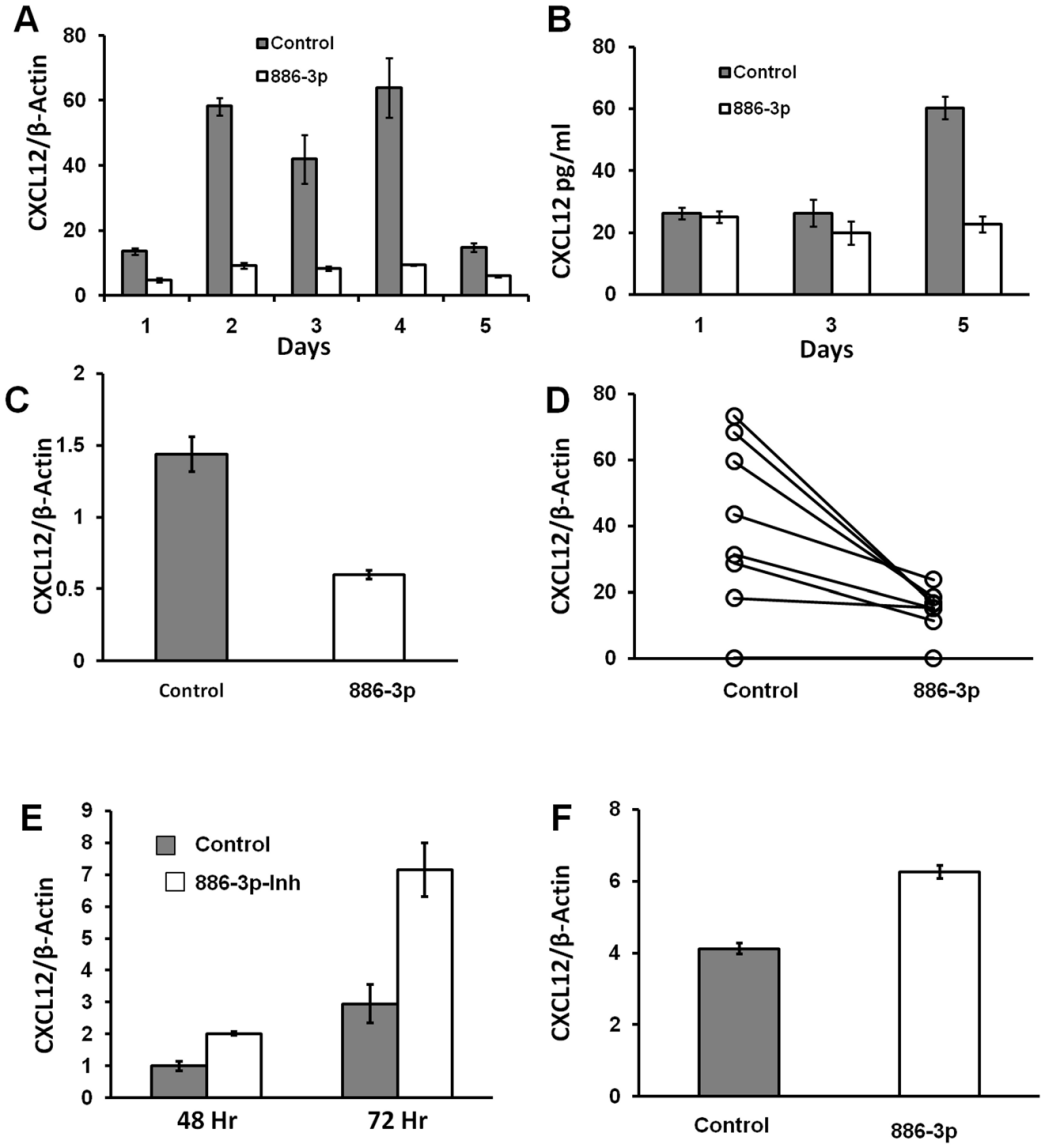
Regulation of CXCL12 by miR-886-3p. **A**: Time course of CXCL12 mRNA expression for 5 days after miR-886-3p compared to control miRNA mimic transfection, as determined by q RT PCR. Results normalized to expression of a house keeping gene beta-actin. CXCL12 expression in cells transfected with miR-886-3p was significantly different from control transfected cells on all 5 days (p<0.05). Results shown as mean of three samples ± SE. **B**: Time course of secreted levels of CXCL12 in conditioned media of HS27a transfected by miR-886-3p or control miRNA as determined by quantitative ELISA. A standard curve was constructed using dilutions of recombinant CXCL12 to calculate concentration in individual samples. Results expressed as pg/ml, and are the mean of three samples, ± SE. CXCL12 levels were significantly different (p<0.05) in miR-886-3p transfected and control transfected media only on day 5. **C**: CXCL12 gene expression in HS27a cells after stable transfection of miR-886-3p primary transcript (363 bp in length) by a gamma-retro viral vector. Control transfections had no insert at the corresponding site. Results expressed as a ratio to beta-actin expression, p value <0.05. **D**: CXCL12 gene expression in 8 primary fibroblast cultures (derived from LTCs) after 48 hours of transfection with miR-886-3p or control miRNA. Paired samples are connected by a line. Statistical analysis performed by Wilcoxon matched pair test, p value was <0.05. **E**: Up regulation of CXCL12 in HS5 cells after down-regulation of miR-886-3p at 48 and 72 hours after transfection. MiRNA antagonists (locked nucleic acid or LNA probes against miR-886-3p or a negative control) were transfected by reverse transfection; (p<0.05 at 72 hours). **F**: CXCL12 gene expression in HS5 cells after stable transfection of “miR-886-3p sponge” primary transcript (363 bp in length) by a gamma-retro viral vector. Control transfections had no insert at the corresponding site.(p value <0.05).

**Figure 4:**
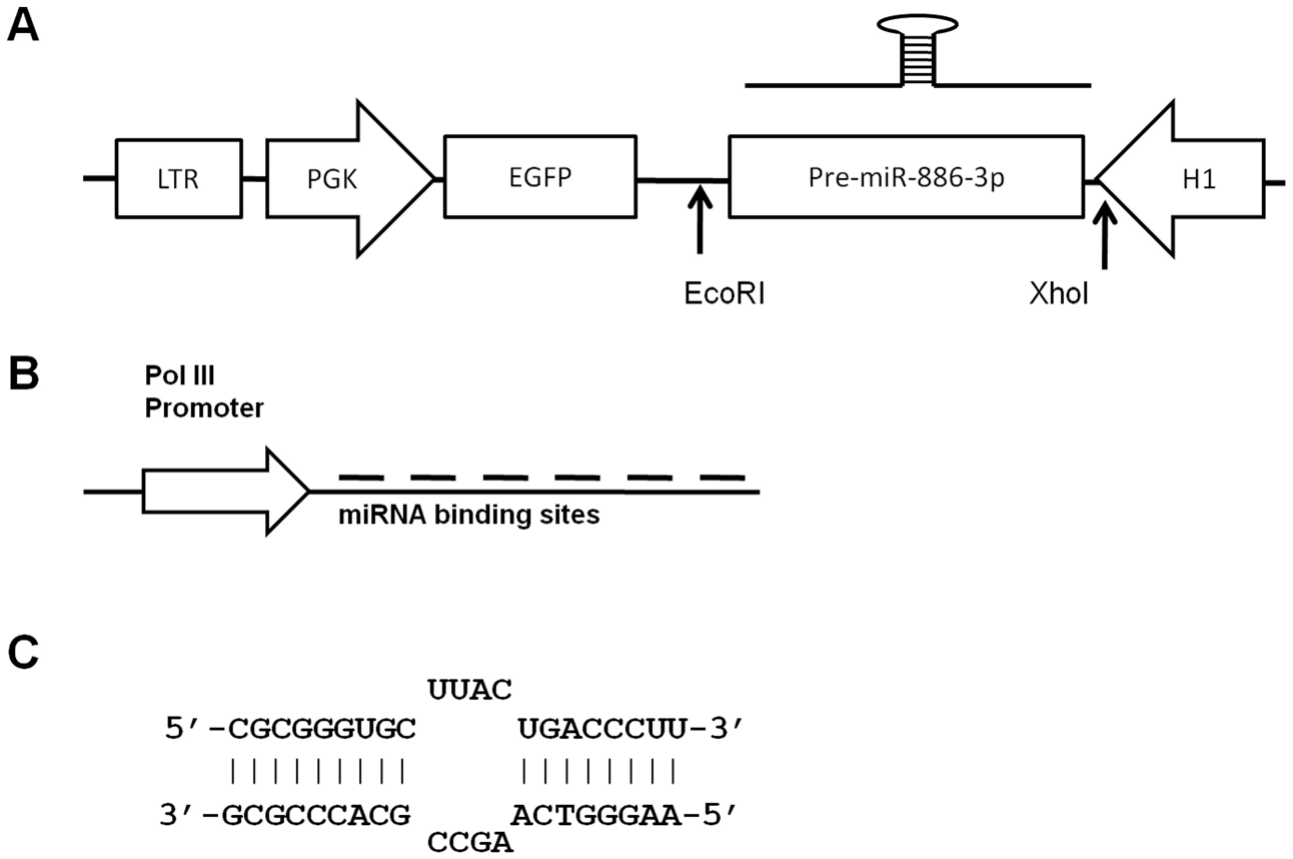
Stable Expression of miR-886-3p or its knock-down using viral vectors. **A**: MDH1-PGK-EGFP retroviral vector for the stable expression of miR-886-3p and miRNA sponge. The constitutively active PGK promoter drives the expression of EGFP while the Pol III promoter H1 drives the expression of the 363 bp genomic DNA fragment that includes the primary transcript, or the miRNA sponge. **B**: The miRNA sponge. A 240 bp transcript with 8 repeats of 21 mers partially homologous to miR-8863p was synthesized and cloned into the EcoRI-XhoI site of MDH1 vector. **C**: Sequence of miRNA sponge. A “bubble” region is included as per initial reports that partial non-homology improves the binding and down-regulation of the miRNA.

The select mRNAs quantified included Jagged-1(JAG1), Angiopoietin1 (Ang1), CXCL12, Bone Morphogenetic Protein4 (BMP4), N-Cadherin (CDH2), VCAM-1 and Frizzled 4(FZD4). Amongst these, CXCL12 was found to be markedly down regulated by miR-886-3p transfection when compared to controls. The other genes had insignificant changes in expression level (data not shown). **Figure 3A** shows that the maximum down regulation of CXCL12 was attained on Day 4 when levels falls as much as 85% of control. Protein expression was also measured by ELISA. As shown in **Figure 3B**, at day 5 post transfection the levels of secreted CXCL12 were significantly reduced in supernatants from cells transfected with miR-886-3p compared to controls. We also achieved the stable over-expression of miR-886-3p achieved by cloning a 363 bp fragment that includes the 121bp primary transcript using the MDH1-PGK GFP retroviral vector [25] (as obtained from Chang-Zhen Chen's laboratory at Stanford University through Addgene, plasmid #11375). This strategy also resulted in reliable down-regulation of CXCL12 in HS27a cells (**Figure 3C**). The retroviral vector is represented in **Figure 4A**.

The down-regulation of CXCL12 was also seen in primary stromal fibroblasts obtained from long term cultures (LTCs). Despite variability in the basal expression level of CXCL12, 6 of 8 primary stromal cultures also showed significantly decreased expression of CXCL12 at 48 hours after transfection (**Figure 3D**). We also confirmed the down regulation of CXCL12 by miR-886-3p in non-stromal cell lines (U87MG, A375, T47D and MCF7, data not shown).

Further evidence for the association of miR-886-3p with CXCL12 expression came from complementary experiments in which the expression level of miR-886-3p was knocked down in HS5 cells by transient transfection of anti-sense locked nucleic acid (LNA) probes. Results are shown in **Figure 3E**. Using the same MDH1-PGK-GFP retroviral vectors described above, we then proceeded to perform stable knock-down of miR-886-3p in HS5 cells by the expression of “microRNA sponges”. miRNA sponges are oligonucleotides consisting of 8-fold repeats of antisense-sequence to the particular miRNA, which acts like a sponge to “mops-up” the free miRNA [26]. HS5 cells with stable-knock-down of miR-886-3p had increased CXCL12 expression comparable to that obtained with transient knock-down using LNA-probes. Details of the miR-886-3p sponge are shown in **Figure 4B and 4C**. Taken together, we conclude that miR-886-3p is a regulator of CXCL12 expression in marrow stromal cells.

### CXCL12 3′UTR is a direct target of miR-886-3p

**Figure 5:**
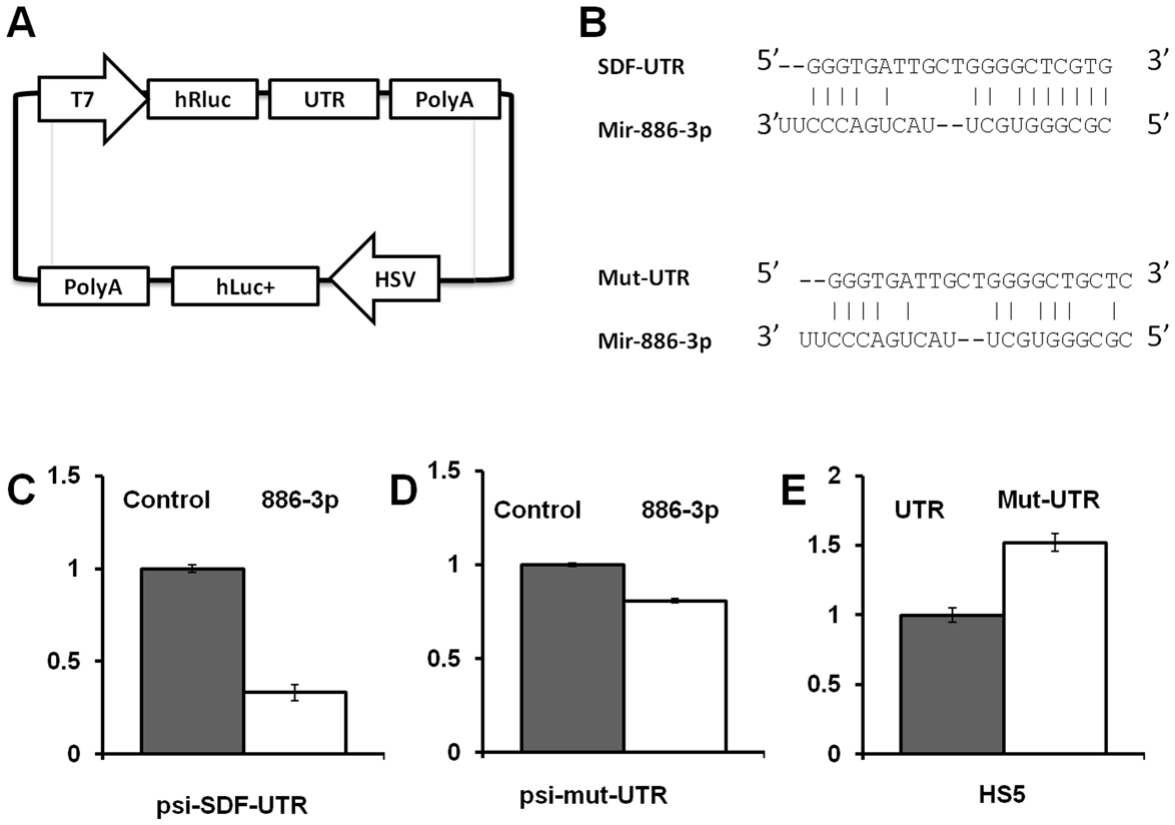
Analysis of effect of miR-886-3p on 3′UTR of CXCL12. **A**: psi2-CXCL12-UTR vector with both firefly luciferase (fLuc+) as internal control and Renilla luciferase (hRluc) upstream of the UTR construct. **B**: Putative binding site of miR-886-3p on the CXCL12 UTR along with the 3 nucleotide mutation in the seed region (termed Mut-UTR). **C**: Luciferase assay for psi-SDF-UTR in HS27a cells when co-transfected with miRNA control mimic or miR-886-3p. Results normalized to control transfections (with irrelevant miRNA mimic) which is assigned a value of 1, p<0.05. **D**: Luciferase activity of psi-mut-UTR in HS27a cells when co-transfected with miRNA control mimic or miR-886-3p. **E**: Luciferase activity of psi-UTR or psi-mut-UTR in HS5 cells, no miRNA co-transfection, p<0.05. All luciferase measurements were made in triplicates and readings were performed at 48 hours after transfection are represented as ± SE.

To determine if CXCL12 mRNA is a direct target for miR-886-3p binding, we cloned the full length 3′ UTR of CXCL12 from genomic DNA and inserted it downstream of the renilla luciferase gene in the psi-Check2 plasmid (**Figure 5A**) to create the psi-SDF-UTR plasmid. The psi-check2 plasmid has both the firefly luciferase and renilla luciferase expressed from constitutive promoters. The 3′ UTR cloned immediately downstream of the Renilla luciferase gene is expected to modulate the expression of Renilla luciferase alone; firefly luciferase serves as an internal control to which the expression of renilla luciferase can be normalized [27]. Stromal cells were co-transfected with miR-886-3p or the control plasmid and analyzed for expression at 48 hours using a dual luminometer. When the 886-3p mimic was co-transfected with psi-SDF-UTR we detected a 70% reduction in renilla luciferase expression compared to control (**Figure 5C**). This suggests that the predominant effect of miR-886-3p in decreasing CXCL12 expression is mediated through its 3′UTR.

To identify the miR-886-3p binding site within the UTR we initially used the target prediction program (miRANDA and miRBase). However these programs did not reveal any putative binding sites presumably due to the need for cross-species validation in their algorithms (miR-886-3p has only been described in humans and macacus mulata). We then used the RNA22 algorithm (http://cbcsrv.watson.ibm.com/rna22_targets.html) [27], which did show alignment of the putative seed region within the UTR. Three nucleotides of the putative 7 nucleotide seed region were then mutated as shown in **Figure 5B** by site directed mutagenesis to disrupt the binding of miR-886-3p. This mutation reduced the suppressive effect of miR-886-3p on the luciferase activity when compared to the unmutated UTR, as shown in **Figure 5D**. Further evidence for a direct effect of miR-886-3p on the CXCL12 UTR came from analysis of the 3′ UTR in HS5 cells. As HS5 has high endogenous expression of miR-886-3p, plasmids were transfected alone without transfection of additional miRNA precursors. As shown in **Figure 4E**, mutating the putative binding site on the UTR increased luciferase activity significantly, again suggesting that the down regulation of CXCL12 is effected by miR-886-3p through direct binding to the putative binding site.

### miR-886-3p transfection of HS27a decreases chemotaxis of Jurkat cells

**Figure 6:**
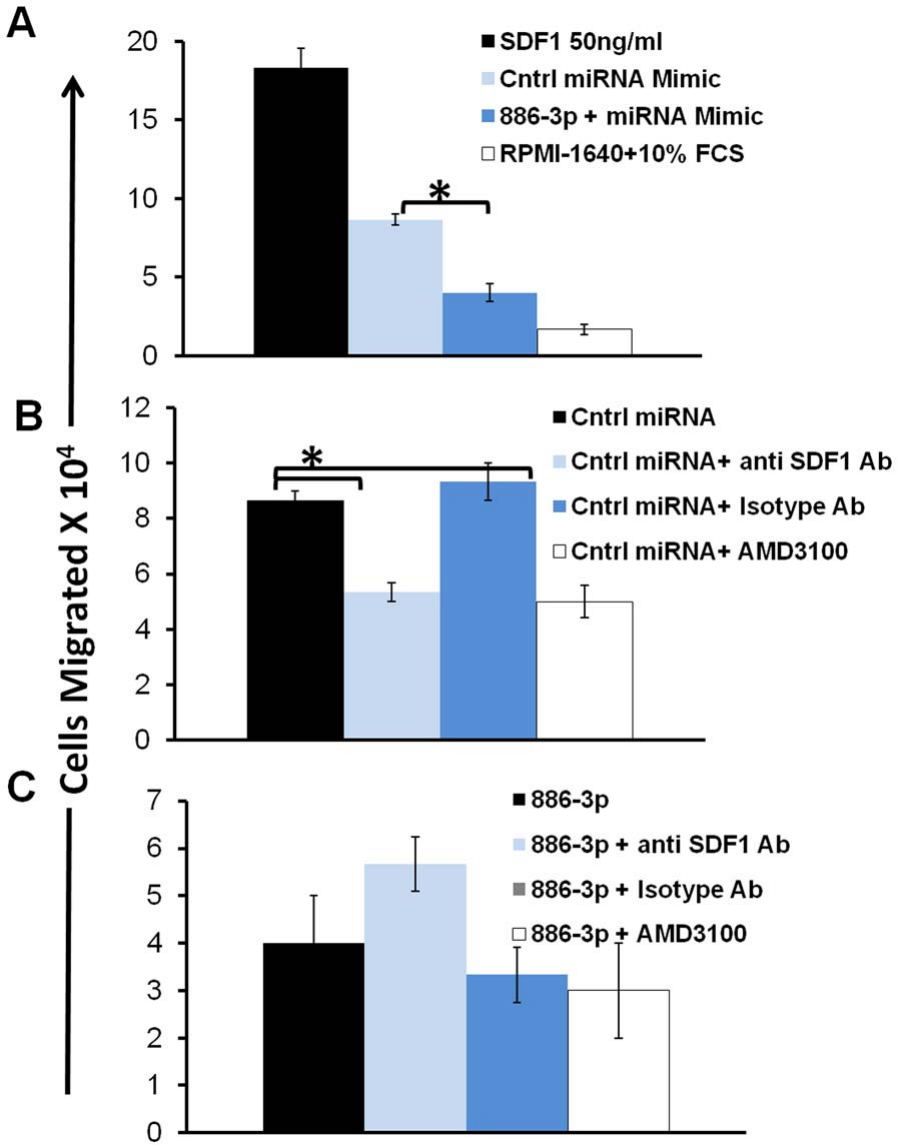
Chemotaxis Assay with Jurkat Cells. **A**: Chemotaxis of Jurkat cells in response to Control miRNA mimic transfection or mir-886-3p transfection of HS27a cells. Media was collected after 5 days of transfection and used for chemotaxis assay. Controls included CXCL12 (50 ng/ml) and RPMI1640 with10% FCS. **B**: Chemotaxis of Jurkat cells to control mimic transfected HS27a media. Controls include the same media treated with neutralizing anti-SDF1 antibody, or mouse IgG1 antibodies at 100 ug/ml, or pre-treatment of Jurkat cells with 1000 ng/ml of AMD3100. The neutralizing antibody or AMD3100 reduced the chemotactic response of Jurkat cells. **C**: Chemotaxis of Jurkat cells to mir-886-3p transfected HS27a media. The same controls are used as in Panel B. Treatment with inhibitors (neutralizing antibody or AMD3100 failed to change the chemotaxis of Jurkat cells. All samples were done in triplicates. Statistical comparisons of relevance that reach significance by student t-test (p<0.05) are indicated by “*”.

To determine the functional significance of miR-886-3p mediated down regulation of CXCL12, we measured the effect of transfected mir-886-3p on the chemo-attractant activity of HS27a conditioned media. The Jurkat T-lymphoid cell line has a well documented chemotactic response to CXCL12 [28], which is secreted by HS27a (**Figure 2B**). In these experiments conditioned media from HS27a cells transfected with miR-886-3p or with controls miRNAs, were collected 5 days after transfection and placed in the lower chamber of a trans-well. Jurkat cells were plated in the upper chamber separated by a porous membrane (5 µ pore size). The migration of Jurkat cells to the lower chamber was quantitated after 3 hours, per published methodology [28]. In addition to CXCL12, HS27a conditioned media is likely to have other molecules with chemo-attractant properties, hence several controls were included; CXCL12 at 50 ng/ml as a positive control and RPMI 1640 with 10% fetal calf serum as a negative control. Results shown in **Figure 6A** indicate that transfection of miR-866-3p into HS27a cells significantly reduced the ability of HS27a conditioned media to support Jurkat chemotaxis. As positive controls, there was a partial inhibition of the chemotactic effect by both a neutralizing antibody to CXCL12 or pre-treatment with a small molecule inhibitor of CXCR4, AMD3100 [29] (**Figure 6B**). Importantly the reduced chemo-attractant potential of conditioned media from miR-886-3p transfected HS27a cells was not further reduced by addition of either the neutralizing antibody or AMD3100. This suggests that the conditioned media from the miR-866-p3 transfected HS27a cells was already depleted of CXCL12 (**Figure 6C**). Taken together, CXCL12 mRNA down regulation by miR-886-3p inhibits function attributable to CXCL12.

## Discussion

Mesenchyme-derived stromal fibroblasts (also referred to as marrow stromal cells, mesenchymal stem cells, or MSC) are now known to play a critical role in the regulation of hematopoiesis as defined by both in vivo and in vitro models [16]. Although the term MSC has come to imply that these cells are homogenous, it is now recognized that the MSC population contains subsets with distinct functions, although isolating these subsets by surface labeling has been difficult [30]. Our group has previously used the STRO-1 antibody to isolate the bone marrow mononuclear cells (BMMNC) capable of forming colony forming unit fibroblasts (CFU-F) [31]. Recently, CD146 or MCAM was used by Sacchetti et al to define the subset of stromal fibroblasts that contribute to a stem cell niche. In this study, we have used functionally distinct stromal cell lines that have been extensively characterized for their ability to influence hematopoiesis. And in agreement with Sacchetti' et. Al's observations, the line that supports progenitor cells in their undifferentiated state, HS27a, expresses high levels of CD146. This is in contrast to a second line, HS5, isolated from the same primary culture but significantly different in function.

Little is known as to how distinct functional compartments are specified within this compartment of stromal cells. Trans-activating factors such as transcription factors and microRNAs, a newly described class of small non-coding RNAs are likely involved in this regulation. MicroRNAs (miRNAs) are a class of small (usually ≈22 nucleotides long) non-coding RNAs whose importance in regulating diverse genes has been only recently identified [32], [33]. MiRNAs are transcribed as primary transcripts of variable length or arise from intronic regions of mRNAs, and are then serially processed enzymatically by the Drosha and Dicer complexes to generate the mature miRNA [34]. They are now known to negatively regulate gene expression by binding to the 3′ untranslated region (3′UTR) of mature mRNAs in a sequence-specific manner and repressing gene expression by either initiating degradation of the mRNA or by preventing it's translation to a peptide[35].

In this report, we provide evidence that CXCL12, a critical component of the stem-cell niche is regulated, at least in part, by miR-886-3p. This miRNA has been previously described by deep sequencing of small RNA libraries and is most abundant in connective-tissues, but not demonstrated by hybridization to a size-fractionated RNA sample (such as Northern Blotting), which is considered a pre-requisite to denoting a small RNA as a microRNA[36]. In addition, recent reports have suggested that the primary transcript of miR-886-3p may be the RNA component of a ribonucleoprotein (RNP) called vault [15] and that the transcript may not be a miRNA [20]. Hence we first sought to demonstrate that the 121-nuceotide-long primary transcript and the 21 nucleotide mature miRNA could be detected in the stromal cell lines by northern blotting. In addition to direct demonstration of the miRNA, our results extend the recent observations that small regulatory RNAs may be derived from non-coding RNA precursors that may have alternative functions (in this case, as part of a ribonucleoprotein).

The profound down-regulation of CXCL12 by miR-886-3p along with its up-regulation when the miRNA is knocked down suggests that this miRNA is a major regulator of CXCL12. Interestingly, several prediction algorithms (miRBASE, miRANDA and target scan) failed to predict the binding of miR-886-3p's seed region to the 3′ UTR of CXCL12. However a binding site was predicted by the RNA22 algorithm which does not rely on cross-species conservation of binding sites for prediction. These results highlight the limitations of current bioinformatic prediction algorithms for identifying miRNA-mRNA binding of potential significance [37], [38]. Using mutation studies of the putative binding site predicted by RNA22 we were able to confirm that the CXCL12 3′ UTR is a direct target of miR-886-3p.

The regulatory properties of miRs on diverse genes (over 30% of human genes are now thought to be directly regulated by miRs [39]) has added an additional layer of complexity to our understanding of the regulation of gene expression. The chemokine CXCL12 and its receptor CXCR4 are now known to be critical for the homing of HSC and their progeny to appropriate niches in the bone marrow [40]. CXCL12 is also known to be secreted by diverse cell types, in the hematopoietic ME, stromal cells (termed CXC12 abundant reticular cells or CAR) are thought to be its major source. MiR-126 [41], [42] and miR-23a [43] have been previously reported to regulate the expression of CXCL12. Interestingly, these miRNAs were not expressed differentially between the two stromal cell lines that we interrogated. Cytokines such as IL1beta[12] and FGF2[13],[44] are now known to be associated with the down regulation of CXCL12, but interestingly, neither of these cytokines have a significant effect on the CXCL12 promoter[44],[45]. In fact, FGF2 has been shown to affect the stability of CXCL12 mRNA [44]. Our results suggest that miR-886-3p may play a critical role in CXCL12 expression. As CXCL12 is known to direct homing in diverse tissues and play a role in tumor metastases, it is likely that miR-886-3p expression is likely to be of significance in the regulation of both normal and disease tissue.

Although the coordinated expression of several genes by several different cell types is required to define a functional stem cell niche, the regulation of this coordinated expression is not well understood. Defining the role of miR-886-3p in stromal expression of CXCL12 raises the possibility that regulatory RNAs may play a wide role in regulating the hematopoietic niche. Identifying the trans-acting factors, including transcription factors and miRs, involved in regulating the hematopoietic ME would facilitate the identification of therapeutic targets for diseases such as myelodysplastic syndrome (MDS) in which the marrow ME itself is known to be dysregulated[46],[47]. The recently described techniques of cross-linked immunoprecipitation (CLIP) [48] which relies on pull-down of RNA-argonaute complexes by immunoprecipitation should aid in a system-wide dissection of miRNA-mRNA interactions of functional significance, which was previously limited to bioinformatics based analysis.

## Materials and Methods

### Cell culture, FACS analysis, and sorting

All samples from human volunteers used in the study were obtained in accordance with protocols approved by the Institutional Review Board (IRB) at the Fred Hutchinson Cancer Research Center (FHCRC) and the University of Colorado. Human Stromal cell lines HS5 and HS27A and Jurkat cells were grown in RPMI-1640 supplemented with 10% Fetal Calf Serum (FCS) and Penicillin (100 U/ml and streptomycin (100 µg/mL). A375, T47D and MCF7 cells were grown in DMEM supplemented with 10% FCS and Penicillin-Streptomycin. Jurkat, U87MG, A375, T47D and MCF7 cells were obtained from ATCC (Manassas, VA). Primary stromal fibroblasts were cultured from bone marrow mononuclear cells (BMMNC) as previously described [49]. Cells were stained with FITC-conjugated anti-CD146 antibody (Ebiosciences, San Diego, CA) as well as appropriate isotype control for analysis as well as cell sorting in to CD146^hi^ and CD146 ^lo^ populations on a FACS Aria cell sorter (BD Biosciences, San Jose, CA).

### Transfection of miRNA mimics and inhibitors and retroviral transfection for stable cell lines

Both stromal cell lines and primary stromal cells were transfected by “reverse transfection”(cells were added after the lipid-RNA complex) with Lipofectamine 2000 reagent (Invitrogen, Carlsbad, CA) in a 12 well format. Other cells were transfected with regular lipofectamine 2000 transfection per manufacturer's instructions on adherent cell cultures, reagents volumes and cell numbers were as described for stromal cells. For miRNA over-expression, miRNA mimics from Dharmacon were used at a concentration of 10 nmol along with negative control mimics. For stable expression of miRNAs, a 363 bp genomic region that included the primary transcript of miR-886-3p was cloned into the BamHI-EcoRI site of the previously described MDH1-PGK-GFP vector (Addgene plasmid 11376). Control vectors had no insert. Positive cells were selected for GFP expression by FACS-sorting and single cell colonies were isolated by ring-cloning. MiR-886-3p was confirmed by quantitative RT-PCR. For transient miRNA knock-down, locked nucleic acid (LNA) probes along with negative controls (Exiqon, Denmark) were used at a concentration of 50 nmol. Stable knock-down was attained by the use of “microRNA sponges” expressed using the MDH1-PGK-GFP vector, the sponge was designed based on previous reports[26] and custom synthesized by Genscript (Piscataway, New Jersey) with BamHI and EcoRI sites at the end. Cells were sorted and cloned as for the over-expressing cells. Details of the retroviral vectors and the miRNA sponges are shown in **Figure 4**. Sequence of miRNA sponge and primers used are listed in **Table S2**.

### RNA preparation, miRNA microarray, Northern Blotting and quantitative RT-PCR

For purposes of microarray analysis and quantitative RT-PCR, total RNA was prepared using the miRNEasy kit (Qiagen, Valencia, CA) per manufacturer's instructions; on-column DNAse digestion was performed to eliminate contamination with genomic DNA. For northern blotting, total RNA was prepared using Trizol reagent (Invitrogen) per manufacturer's instructions. MiRNA microarray was performed by Thermo Scientific Dharmacon (Lafayette, CO) on three samples each of HS27a and HS5 and analyzed using textbook ANOVA. For northern blotting, total RNA was separated on a 12% polyacrylamide/8M urea gel on a Protean II apparatus (Biorad, Hercules, CA) and transferred to positive charged nylon membrane using Trans-Blot semi-dry electrophoretic cell (Biorad). The nylon membrane was UV cross-linked and baked at 80°C and probed with P-32 labeled antisense LNA probes (Exiqon). The membrane was then washed and exposed to X-ray film for varying lengths of time.

MiRNAs were quantitated by SYBR-GREEN based quantitative Real Time-Polymerase Chain Reaction (q RT-PCR) on cDNA generated by reverse transcription using a specific stem-loop primer as previously reported. Expression of miR-886-3p was expression was normalized between different samples based on expression of U48 (SNORD48; NR_002745), a small nuclear RNA. Intron-spanning primers were used to quantify CXCL12 mRNA expression and normalized to beta-actin, also by SYBR-GREEN based q RT-PCR assay. Conditions for PCR were 95 degrees for 10 seconds and 60 degrees for 1 minute, for 40 cycles. All products were sequence verified by either direct sequencing of the DNA product or cloning after TA cloning.

### ELISA for CXCL12 levels

Enzyme Linked Immune Sorbent Assay (ELISA) for secreted CXCL12 levels were performed on conditioned media from stromal cells using the Duoset ELISA Development kit (RnD Systems, Minneapolis, MN) per manufacturer's instructions and read on the Synergy micro plate reader (Biotek, Winooski, VT) at 450 nm. A standard curve was constructed using the provided standard for quantification of optical density (OD) values for individual samples.

### DNA Constructs and Luciferase Reporter Assay

The 1.5 Kbp length 3′ UTR of CXCL12 (NCBI Reference NM_199168) was cloned from human genomic DNA with high-fidelity Phusion Polymerase (New England Biolabs, Beverly, MA and cloned into psiCheck2 vector (Promega, Madison, WI). To generate mutants of binding sites, site directed mutagenesis was performed using Pfu Turbo Polymerase (Stratagene, Lajolla, CA) and primers designed by Stratagene's Quikchange Primer Design Program (http://www.stratagene.com/sdmdesigner/default.aspx). All clones were sequence-verified by DNA sequencing. For the dual luciferase assay, 0.4 ug of plasmid was co-transfected with 10 nmol 886-3p mimic or control mimic in 12 well plates as described for transfection with the miRNA alone. At 48 hours, the cells were lysed and assayed for both renilla and fire-fly luciferase using the Dual Luciferase Reporter Assay kit on a GLOMAX micro plate luminometer (both from Promega).

### Chemotaxis Assay

The assays were performed as previously described by Cherla et al [28]. Briefly, Jurkat cells in log-growth phase were washed twice in PBS and 10×10^6^ cells were suspended in RPMI 1940 with 2.5% FCS. The assays were performed in 24 well plates with 5 µm porosity inserts (Corning Inc, Corning NY). 600 µl of conditioned media (or media with antibody) was loaded to the lower chamber, 100 µl of the cell suspension (total of 1×10^6^ cells) was added to the upper chamber. To some wells, mouse anti-human CXCL12 neutralizing antibody (clone 79014) or control mouse IgG1 antibody (both RnD Systems) were added at 100 ug/ml, the ND_50_ of this neutralizing antibody has been determined to be 30 to 60 ug/ml. Some wells had the cells treated with the CXCR4 receptor inhibitor AMD3100^22^ (Sigma Aldrich, St Louis, MO) at 1000 ng/ml concentration for 30 to 60 minutes to block the CXCR4 pathway. Recombinant human CXCL12 (RnD Systems) was added to some wells at 50 ng/ml as positive control. Plates were incubated for 3 hours at 37 deg C in a humid Tissue Culture chamber at 5% CO2, the inserts were then removed and the viable cells in the bottom chamber quantified with a hemocytometer. All wells were repeated in triplicates.

### Statistical Analysis

Textbook ANOVA was used in the differential expression analysis of HS5 vs. HS27A. Student t-test or Wilcoxon matched-pair test was used to determine statistical significance using GraphPad Prism software (La Jolla, CA), a p value of less than 0.05 was deemed statistically significant.

## Supporting Information

Table S1 Microarray analysis of HS27a and Hs5 cell lines.
(0.05 MB XLSX)

Table S2 Primers used in various experiments described in the manuscript.
(0.05 MB XLS)
